# Impact Resistance of Layered Aramid Fabric: A Numerical Study on Projectile-Induced Damage

**DOI:** 10.3390/polym16243522

**Published:** 2024-12-18

**Authors:** Larisa Titire, Cristian Muntenita, Mariana Chivu

**Affiliations:** 1 Faculty of Engineering, “Dunărea de Jos” University, 800008 Galati, Romania; larisa.chiper@ugal.ro; 2 Faculty of Entrepreneurship, Engineering and Business Management, National University of Science and Technology Politehnica, 060042 Bucharest, Romania; mariana.chivu0608@upb.ro

**Keywords:** aramid, impact ballistic, 9 mm Luger, .357 Mag FMJ, NIJ Standard 0123.00

## Abstract

The aim of this work is to comparatively analyze, using numerical simulation, the impact behavior of aramid fabric. A layered panel was impacted by two projectiles specific to the NIJ protection level HG1. The protection level in this study is based on NIJ Standard 0123.00. This standard is used to establish protection levels. The two projectiles specific to the NIJ HG1 protection level are 9 mm Luger and .357 Mag FMJ. Law enforcement personnel use body armor designed to protect the torso. With the help of numerical simulation, the mechanisms of destruction of the aramid fabric on impact are identified. The protection performance is analyzed as a function of the influence of the number of layers and the projectile velocity variation. The fabric is modeled at the yarn level, with each yarn consisting of hundreds or even thousands of fibers. Simulations are performed at the yarn level, since fiber-level modeling is difficult to implement due to high computational resource requirements. The material properties for the yarn, as well as for the projectiles, are selected from the literature. The results show that only the 20-layer fabric panel impacted by the 9 mm Luger FMJ RN 9 mm FMJ RN projectile at 398 m/s meets the protection requirements of the NIJ standard (NIJ HG1 protection level). In contrast, panels impacted at 436 m/s, or those with fewer layers, show rapid stress wave propagation, severe deformation, and complete perforation, indicating insufficient energy dissipation. This study highlights the critical role of impact velocity, projectile geometry, and number of layers in determining ballistic resistance. These findings contribute to the development of more effective ballistic protective equipment, highlighting the need for optimized layer configurations and improved material properties to meet NIJ standards under different impact conditions.

## 1. Introduction

As high-performance computing systems become more accessible, the use of computer simulation in studying the ballistic performance of high-strength fabric is increasing. When we accurately and thoroughly model the fabric system, simulations can yield valuable data that experimentation cannot provide [1,2,3,4,5,6,7,8].

These simulations allow for detailed investigation of the impact behavior of materials, providing information on stress distribution, fabric deformation, and energy absorption mechanisms [9,10,11]. In addition, simulations can explore a wide range of impact scenarios and design parameters, saving time and resources compared to experimental tests. As a result, they have become an essential tool in the development and optimization of high-performance ballistic materials [12].

Protecting defense personnel is always a priority for protection against enemy weapons while ensuring sufficient mobility. In recent years, advances in weaponry have necessitated a significant increase in protective measures, resulting in armor that has become increasingly cumbersome. This has negatively impacted on soldier mobility [13]. Due to their low weight and high strength to weight ratio, polymer matrix composites are widely used in sporting goods, aerospace vehicles, and ballistic applications [14]. Contemporary armor systems employ various polymeric materials to provide superior ballistic protection while providing reducing weight and enhancing mobility for both the users and the vehicle [15].

Aramid fibers are very desirable because of their lightweight nature, exceptional specific strength, and superior stiffness compared to other materials, such as metals [16]. Furthermore, their exceptional damage tolerance and failure mechanisms contribute to their remarkable ballistic impact performance and their ability to absorb significant amounts of energy [17]. As a result, aramids are extensively used in the fabrication of structural impact and protective systems across various domains, primarily for safeguarding individuals, such as battle helmets and bulletproof vests, in the military sector.

The mechanical properties and interaction mechanisms of the individual fabric components at all levels of their structure (fibers, yarns, fabric layers, and ballistic inserts) contribute to the high energy absorption and dissipation capabilities of para-aramid fabrics. Their experimental findings are often complemented by numerical calculations due to the complexity of the systems that influence textiles’ behavior in projectile impact situations. Simulations offer crucial insights into the process and help to enhance understanding of the event.

Numerical modeling of ballistic panels is carried out in the literature on three levels. These levels represent layers of fabrics at three different levels: fiber level (microstructural), strand level (mesostructural), and layer level (macrostructural). Each level has unique advantages and disadvantages. Layers of fabrics composed of solid components are referred to as uniform membranes at the macrostructural level [18,19]. The characteristics of such a layer are described using both anisotropic [20,21] and isotropic [22] material models. This modeling approach has a low computational cost since it ignores the geometry and entanglement of the yarns. As a result, impacts can be modeled on ballistic inserts with multiple layers of significant size. Energy dissipation through the friction of interacting fibers and yarns is one of many important interaction mechanisms in yarns and fibers that are overlooked, even though it is particularly important in the cases of projectile impact in fabrics and greatly influences their high protection capability [23,24].

Modeling fabrics at the yarn level is the most popular. Compared to the layer-level method, more accurate results can be obtained since such models include the geometry of the yarns as well as the mechanics of their interaction [25,26,27,28]. This allows for the exact reproduction of the interactions between yarns, such as friction [25,26,27,28].

The fabric is described using microstructural techniques as a collection of parallel fibers that are tightly packed in the cross-section of the yarn [29]. As a result, most typical deformation processes and interactions between textile components exposed to both in-plane and transverse stresses can be reproduced. These mechanisms include friction between yarns and fibers, fiber dislocation, and damage and breakage of individual fibers. However, such approaches require high processing power.

A key limitation in current computational models lies in the scale at which the interactions of yarns and fibers are modeled. Many finite element models operate at the mesoscopic scale, which may be too coarse to capture the detailed mechanisms of stress propagation through yarns and the intricate fracture patterns that occur during impact. At the macroscopic level, energy dissipation through mechanisms such as friction between yarns, interfacial bonding between fibers, and the collective behavior of fabric layers often cannot be accurately simulated without significant computational overhead. Consequently, simulations that work well for simple configurations may fail in more complex real-world applications where these mechanisms are critical to fabric performance.

Below are the identified gaps in numerical simulations:

- The models simplify the interaction between the layers, neglecting the friction and comminution between the layers.
- Experimental studies have demonstrated that friction between yarns can significantly increase fabric resistance to ballistic impact, but FEM simulations tend to use constant friction coefficients without accounting for local variations driven by increased pressure or temperature during impact [30,31,32,33,34]. Improved modeling of this phenomenon could lead to more accurate predictions of fabric behavior.
- The fibers may undergo significant stretching, delamination, or rupture, and these mechanisms contribute to the energy-absorbing capacity of the fabric [35,36]. At higher velocities, the impact can cause the fibers to break, split, or even melt in some cases, which is difficult to capture accurately in conventional FEM simulations [37]. Integrating more comprehensive failure models, such as those that include temperature rise due to impact-induced friction, could improve the realism of these simulations.
- Many conventional FEM models assume homogeneity or use simplified isotropic material properties that ignore differences in mechanical behavior between different fiber orientations. This leads to inaccuracies, especially in the case of high-velocity impacts, where fiber orientations significantly influence the way fibers absorb energy. Studies suggest that improved material heterogeneity modeling can lead to more accurate predictions of fabric performance under ballistic impact [38,39,40].
- The impact of a projectile can cause the first layers to compress and absorb energy, but this force is transmitted to deeper layers, where interactions can become complex. These forces, along with friction and deformation mechanisms between the layers, are often simplified or ignored in FEM simulations. As a result, deformation and fault propagation along the layers may not be accurately captured, leading to underestimation or overestimation of fabric performance. More advanced models, such as those incorporating multiscale modeling (e.g., from micro to macro level), could help to address this discrepancy [28,41,42,43,44,45,46].

Before examining ballistic materials in detail, it is necessary to consider the real impact scenario. Many typical characteristics are related to the effect of a projectile on the protection system, regardless of the type of threat (projectile, knife, low/low-velocity pistol bullet [16,47,48,49,50].

- The projectile exerts a significant point load on the protection system.
- The medium-to-high-velocity impact generates stress waves in the material of the protective system, which are also generated on the interior surfaces, resulting in significant secondary effects such as blunt injuries to the rear face of the protective system.
- The impact induces significant deformation stresses on the protection system, resulting in localized damage.
- The material of the protective system exhibits varying responses in distinct impact situations.

The fundamental component of the ballistic fabric is usually a fiber or yarn. Impact analysis of a yarn can provide information about the ballistic impact behavior of fabrics. During impact, two distinct types of waves are generated on a yarn that propagate from the contact zone of the projectile with the fabric [16,51,52,53,54,55,56]. The first type of wave, associated with the stretching of the yarn, is named the longitudinal wave, which propagates along the length of the yarn, propagating outward. The transverse wave is the second type of wave that propagates during impact, characterized by a lower velocity and displacement of the thread material in the direction of impact [41,43,57,58,59,60,61,62,63,64].

Standard NIJ (National Institute of Justice Standard) 0123.00 [65] was developed to be used in conjunction with other standards, such as NIJ Standard-0101.07 [66], for testing and evaluating ballistic personal protective equipment against current ballistic threats that present a life-threatening safety hazard to the wearer. The specifications for projectiles and their velocities in the first edition of NIJ Standard-0123.00 [65] have been revised from Section 2 of NIJ Standard-0101.06 [67] to address current hazards to the wearer (Table 1).

Feito et al. [68], Lim et al. [22], Demircioğlu et al. [69], Zochowski et al. [70], Abtew et al. [16], Nunes et al. [71], and Zhao et al. [72] have all investigated various factors such as fabric layer number, projectile geometry, impact velocity, and material properties, analyzing their effects on ballistic performance, damage mechanisms, and the optimization of protective fabrics and composite materials under ballistic impact conditions.

The aim of this study is to provide a detailed analysis of the behavior of aramid fabric panels under impact from two types of projectiles at different velocities. While the finite element method (FEM) is used via commercial software (“Ansys”, www.ansys.com), the novelty of this research lies in the customization and refinement of the simulation approach to explore specific ballistic phenomena. This study includes detailed stress wave propagation analyses through the fabric layers, focusing on both longitudinal and transverse directions at various time intervals, offering insights beyond standard penetration studies. A refined model for yarn breakage mechanics, which tracks damage progression at the microstructural level, provides a deeper understanding of how individual yarns fail under different impact conditions. Layer-specific performance evaluations investigate the influence of the number of layers, the sequence of failure from the first to the last layer, and the effect of projectile velocity throughout the impact process. Together, these elements contribute to a groundbreaking insight into the dynamic response of aramid panels, extending essential knowledge for optimizing future ballistic protection systems. This study goes beyond conventional analyses focusing on penetration or energy absorption, providing a detailed and comprehensive assessment of both the mechanical behavior and protective effectiveness of ballistic fabrics.

## 2. Materials and Methods

Finite element (FE) simulation is an efficient approach to analyze the mechanisms involved in ballistic impact. The aim of this study is to use the Ansys program to develop a finite element model capable of simulating the transverse impact of a projectile on fabric panels. The model is developed at the level of the individual yarn, keeping the cross-sectional shape of the yarn constant throughout its length. This detailed approach allows for a deeper understanding of the behavior of the materials under impact conditions.

In the context of finite element method (FEM) simulations, equivalent stress (or von Mises stress) is a scalar measure used to evaluate the complex stress state in a material and determine whether it will fail under load. Equivalent stress is used to assess whether the material of aramid panels can withstand projectile impact. It is an effective method for determining stress distribution and stress intensity, identifying critical points where cracking, plastic deformation, or structural failure may occur.

**Figure 1:**
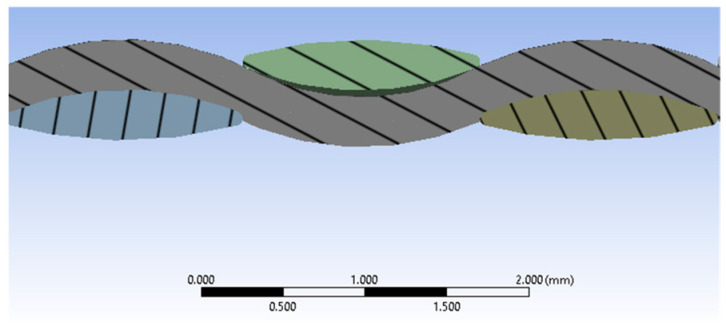
The cross-section of the yarn, as well as the longitudinal section of the yarn.

**Figure 2:**
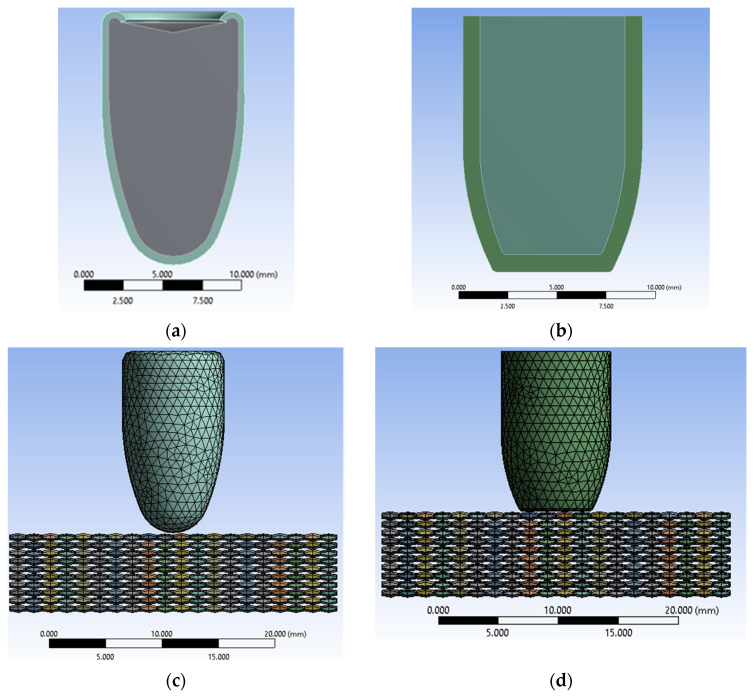
Projectile geometry and model discretization: (**a**) 9 mm Luger FMJ RN projectile geometry; (**b**) .357 Mag JSP projectile geometry. (**c**) Discretization of the ten-layer aramid fabric panel impacted by the 9 mm Luger FMJ RN projectile. (**d**) Discretization of the ten-layer aramid fabric panel impacted by the .357 Mag JSP projectile.

The fabric model used in this study is a simple fabric. The cross-section of the yarn is shown in Figure 1, while the yarn trajectory is the curve describing the unraveling of the yarn as it moves in the opposite direction. Figure 2 shows the panel and projectile geometry and their discretization. The 9 mm Luger FMJ RN projectile has a diameter of 9 mm, a length of 16 mm, and a mass of 7.41 g. The .357 Mag JSP projectile has a diameter of 9 mm, a length of 13 mm, and a mass of 10.24 g [73,74]. The projectiles are deformable. The yarn geometry is described by the cross-sectional shape of the yarn, as well as the trajectory of the yarn. The dimensions of the fabric are 29 mm × 29 mm. The dimensions chosen for fabric layer patterning are those of Twaron CT750 aramid fabric; the thickness of the fabric layer is 0.75 mm, and the yarn width is 1.45 mm. Each layer has 20 yarns in the weft direction and 20 yarns in the warp direction. We used fabric layer solids and projectiles to accurately capture 3D stress distribution and localized deformations under ballistic impact. The yarn and projectile are selected as deformable elements.

For both the fabric and the projectiles, the material properties were selected based on the literature values (references [75,76,77,78,79,80,81,82,83,84,85]) and are given in Table 2, Table 3, Table 4 and Table 5. The yarn material of the fabric is Twaron CT750 aramid, known for its high strength and durability, which is often used in ballistic applications.

To optimize computational time, a symmetry plane was applied to the model. This allows for the reduction in the simulation domain, given the assumption of symmetric loading.

For connections within the model, frictional interactions were defined between the yarns and between the projectile and the fabric layers. A friction coefficient of 0.29 was applied, based on the literature values that are typical for fabric–armor interactions.

The mesh was generated using the Automatic Mesh Method with Linear Element Order. The mesh statistics for different panels and projectiles are provided in Table 6, which outlines the number of nodes and elements for each case, and the mesh size is 0.8.

The initial impact velocity of the projectiles was set to 298 m/s, 398 m/s, and 436 m/s for different scenarios. These velocities were chosen to simulate a range of real-world impact conditions, with 398 m/s and 436 m/s corresponding to higher threat levels, selected from NIJ standard 0123.00.

The fabric layers were constrained using fixed supports at the cross-sectional boundaries of the yarns, which prevents displacement at the edges of the fabric while allowing for deformation and stress propagation within the fabric layers.

The simulations were run using ANSYS Explicit Dynamics to capture the dynamic nature of ballistic impact events. The explicit solver is well-suited for this type of simulation, where time-dependent deformation and material failure occur in a very short period.

**Figure 3:**
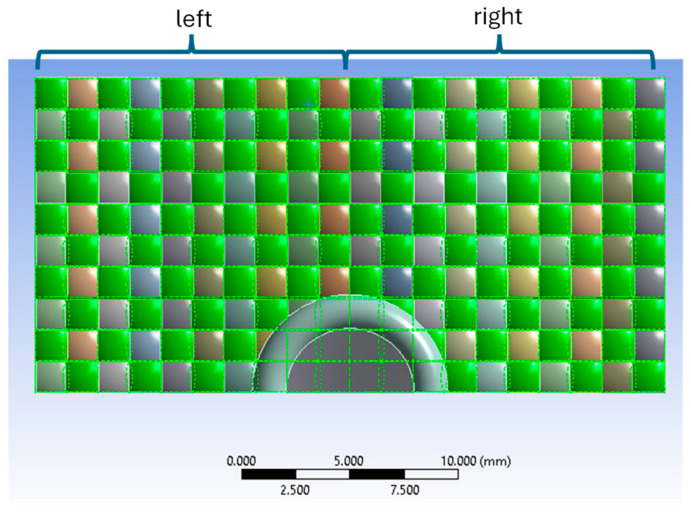
Top view of the panel impacted by the 9 mm Luger FMJ RN FM projectile (the green colored yarns are the yarns in the direction of the warp).

Figure 3 gives the top view of the panel hit by the 9 mm Luger FMJ RN FM 9 mm Luger FMJ projectile (the green colored yarns are the yarns in the warp direction).

## 3. Results

The simulation time is different for the two panels. For the 10-layer panel it is $t=1\times 10^{-4}\;s$, and for the 20-layer panel $t=2\times 10^{-4}\;s$. The initial impact velocities are 298 m/s, 398 m/s, and 436 m/s.

### 3.1. The Influence of Projectile Velocity

#### 3.1.1. Results of the Simulation of the Ten-Layer Fabric Panel Impacted by the 9 mm Luger FMJ RN Projectile with Two Different Impact Velocities: 398 m/s and 436 m/s

**Figure 4:**
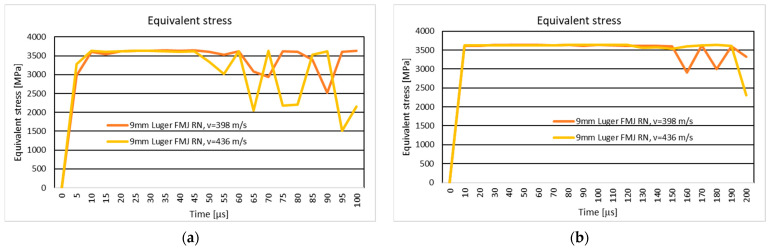
Equivalent stress distribution: (**a**) 10-layer panel impacted by 9 mm Luger FMJ RN; (**b**) 20-layer panel impacted by 9 mm Luger FMJ RN.

Figure 4a shows the equivalent stress distribution during the impact of the stratified panel impacted by the 9 mm Luger FMJ RN projectile.

**Figure 5:**
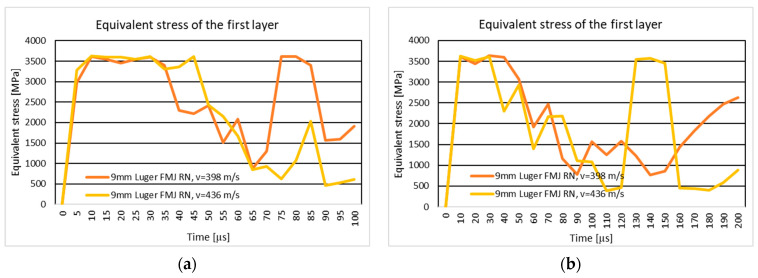
Equivalent stress distribution on the first layer of the panel impacted by the 9 mm Luger FMJ RN projectile: (**a**) 10-layer panel impacted by the 9 mm Luger FMJ RN; (**b**) 20-layer panel impacted by the 9 mm Luger FMJ RN.

Figure 5a shows the equivalent stress distribution during the impact of the first layer of the panel impacted by the 9 mm Luger FMJ RN projectile.

**Figure 6:**
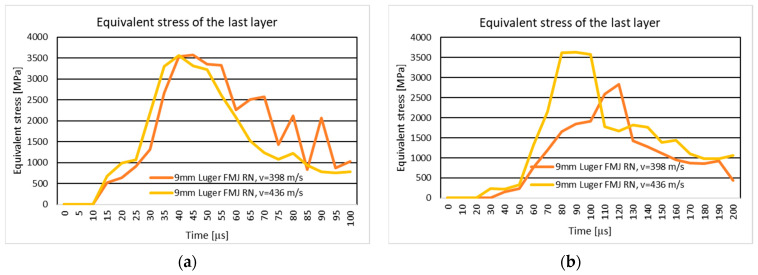
Equivalent stress distribution on the last layer of the panel impacted by the 9 mm Luger FMJ RN projectile: (**a**) 10-layer panel impacted by the Luger FMJ RN; (**b**) 20-layer panel impacted by the 9 mm Luger FMJ RN.

Figure 6a shows the equivalent stress distribution during the impact of the last layer of the panel impacted by the 9 mm Luger FMJ RN projectile.

The moment of time, $t=2\times 10^{-5}\;s$, in both cases, shows stress concentrators on the edge of the yarn, more pronounced in the case with higher velocity. The braking zone is different. In the case of the initial projectile velocity of 398 m/s, the break occurs in the middle of the impact zone, and in the case of the higher velocity, the break occurs between the impact zone (direct contact between the panel and the projectile) and in the area outside the impact. The breaking of the last layers is due to the compression of the first layers and the stretching of the last layers, because the tip of the projectile has not exited the fabric. The projectile is deformed in the tip area.

In the case of a projectile velocity of 436 m/s, the trajectory of the projectile is modified due to the way the yarns break, which is not visible in the other case. The broken fragments in the impact zone are pushed to the left side by the projectile in the case of 436 m/s velocity, and in the case of 398 m/s velocity, the fragments are pushed to both sides.

The braking mode of the first layer is similar. The stress distribution at the time of breakage is also on the main and secondary yarns. The areas with stress concentrators are more scattered in the case with lower velocity.

#### 3.1.2. Results of the Simulation of the Ten-Layer Fabric Panel Impacted by the .357 Mag JSP Projectile with Two Different Impact Velocities: 298 m/s and 436 m/s

**Figure 7:**
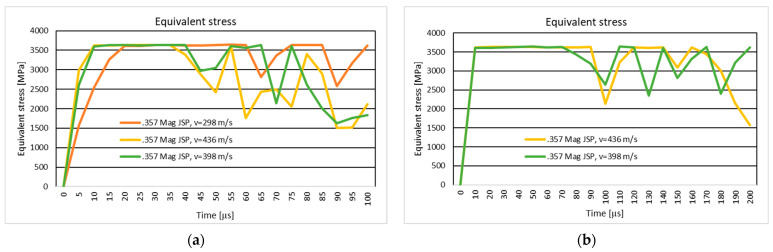
Equivalent stress distribution: (**a**) 10-layer panel impacted by the .357 Mag JSP; (**b**) 20-layer panel impacted by the .357 Mag JSP.

Figure 7a shows the equivalent stress distribution during the impact of the stratified panel impacted by the .357 Mag JSP projectile.

**Figure 8:**
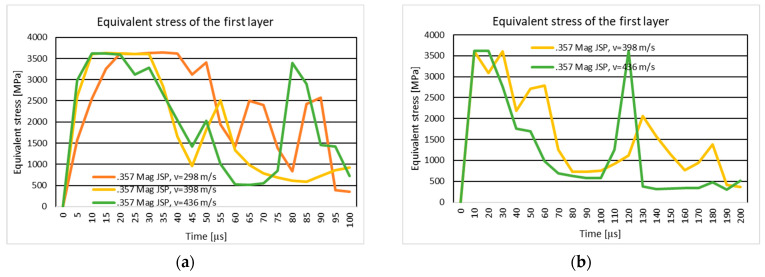
Equivalent stress distribution on the first layer of the panel impacted by the .357 Mag JSP projectile: (**a**) 10-layer panel impacted by the .357 Mag JSP; (**b**) 20-layer panel impacted by the .357 Mag JSP.

Figure 8a shows the equivalent stress distribution during the impact of the first layer of the panel impacted by the .357 Mag JSP projectile.

**Figure 9:**
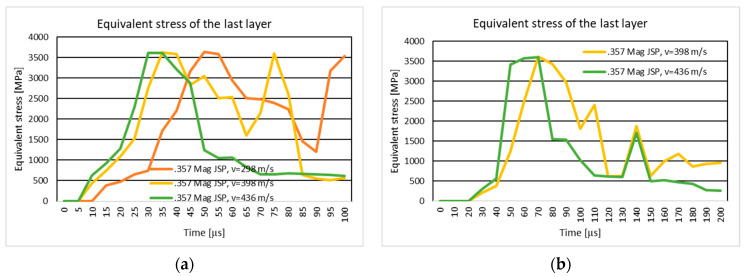
Equivalent stress distribution on the last layer of the panel impacted by the .357 Mag JSP projectile: (**a**) 10-layer panel impacted by the .357 Mag JSP; (**b**) 20-layer panel impacted by the .357 Mag JSP.

Figure 9a shows the equivalent stress distribution during the impact of the last layer of the panel impacted by the .357 Mag JSP projectile.

The moment of time, $t=3.5\times 10^{-5}\;s$, marks the breaking of all layers (broken yarns) without the tip of the projectile exiting the fabric. The breaking of the layers does not take place in the center of the impact zone, but rather takes place to one side, pushing the broken fragments downwards and sideways in the case with a speed of 436 m/s.

The moment of time, $t=6\times 10^{-5}\;s$, marks the breaking of the panel layers. The projectile is not out of the fabric; the breaking of yarns is caused by the compression of the first layers and the stretching of the yarns on the last layers. In the case with the projectile velocity of 436 m/s, the projectile completely exits the fabric. The broken fragments in the layers are thrown to the left side, while in the case with lower velocity, the projectile body does not completely exit the fabric, but all layers are broken. The broken fragments in the layers are on the tip of the projectile. The higher velocity case shows a slight change in the trajectory of the projectile, which also caused the yarns to be thrown to the left side, a phenomenon not visible on the lower velocity case.

#### 3.1.3. Results of the Simulation of the 20-Layer Fabric Panel Impacted by a 9 mm Luger FMJ RN Projectile with Two Different Impact Velocities: 398 m/s and 436 m/s

Figure 4b shows the equivalent stress distribution during the impact of the stratified panel impacted by the 9 mm Luger FMJ RN projectile.

Figure 5b shows the equivalent stress distribution during the impact of the first layer of the panel impacted by the 9 mm Luger FMJ RN projectile.

Figure 6b shows the equivalent stress distribution during the impact of the last layer of the panel impacted by the 9 mm Luger FMJ RN projectile.

At the first time moment, longitudinal stress waves are distributed over more yarns in the case with higher projectile velocity. The time moment, $t=8\times 10^{-5}\;s$, marks the transverse stress wave distribution up to the last layer for the lower velocity case, and the time moment $t=3\times 10^{-5}\;s$ marks the transverse wave distribution up to the last layer.

In the case of the lower projectile velocity, the projectile remains trapped in the fabric, while in the case of the 436 m/s velocities, all layers are perforated and the nose of the projectile comes out of the fabric.

Maximum stress values at the first layer are similar for both velocities, but the higher velocity leads to more severe deformation at later stages, with a higher final stress value recorded.

#### 3.1.4. Results of the Simulation of the 20-Layer Fabric Panel Impacted by the .357 Mag JSP Projectile with Two Different Impact Velocities: 398 m/s and 436 m/s

Figure 7b shows the equivalent stress distribution during the impact of the stratified panel impacted by the .357 Mag JSP projectile.

Figure 8b shows the equivalent stress distribution during the impact of the first layer of the panel impacted by the .357 Mag JSP projectile.

Figure 9b shows the equivalent stress distribution during the impact of the last layer of the panel impacted by the .357 Mag JSP projectile.

The time moment, $t=6\times 10^{-5}\;s$ , records longitudinal stress waves on all layers for the higher velocity case, and in the case of a velocity of 436 m/s, longitudinal stress waves are distributed on all layers at the time moment, $t=5\times 10^{-5}\;s$.

The breakup of the 20 layers is visible at the time moment $t=9\times 10^{-5}\;s$ for the 398 m/s velocity case, and at the time moment $t=5\times 10^{-5}\;s$ for the 436 m/s projectile velocity.

At the end of the simulation, the projectile in both cases changes its trajectory. In the case with lower velocity, the projectile is rotated almost 90 degrees, and the broken fragments of the yarns in the impact zone are pushed in the direction of projectile rotation. The projectile deformation mode is approximately identical.

### 3.2. The Influence of the Number of Layers

#### 3.2.1. Results of the Simulation of the 10-ply Fabric Panel and the 20-ply Fabric Panel Impacted by the .357 Mag JSP Projectile with an Initial Impact Velocity of 436 m/s

**Figure 10:**
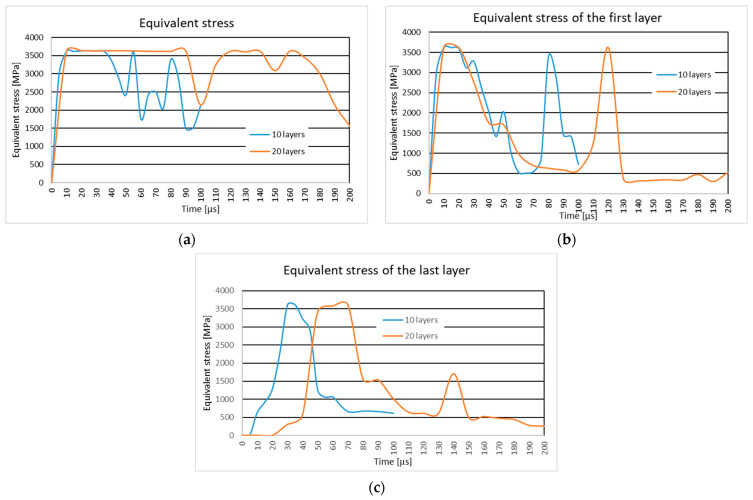
Equivalent stress distribution of the 10-layer fabric panel and the 20-layer fabric panel impacted by the .357 Mag JSP mm projectile with an initial impact velocity of 436 m/s. (**a**) Equivalent stress distribution of the panel. (**b**) Equivalent stress distribution of the first layer. (**c**) Equivalent stress distribution of the last layer.

In Figure 10, the equivalent stress distribution during the impact of the layered panel impacted by the .357 Mag JSP projectile is shown along the equivalent stress distribution on the first and last layer of the 10-layer panel and the 20-layer fabric panel.

Both panels are completely penetrated. The projectile changes its trajectory during the impact, the difference being in the angle it makes with respect to the direction of impact, which is larger for the 20-layer panel. The projectile is more deformed in the 20-layer panel.

The longitudinal stress waves on the first layer are distributed on the main yarns in the case of the ten-layer panel. On the 20-layer panel, the longitudinal stress waves are distributed on both the main and secondary yarns. The stress concentrators are distributed over a larger area in the case of the 20-layer panel (in the impact zone, but outside this zone, the center main yarn in the weft direction has stress concentrators up to the embedding zone).

The time moment, $t=3\times 10^{-5}\;s$, records for the 10-layer panel, the stress concentrators, along the whole length of the main yarns, in the warp direction and in the weft direction. The longitudinal stress waves are being distributed on all the yarns (both main and secondary yarns).

#### 3.2.2. Results of the Simulation of the 10-ply Fabric Panel and the 20-ply Fabric Panel Impacted by the 9 mm Luger FMJ RN Projectile with an Initial Impact Velocity of 436 m/s

**Figure 11:**
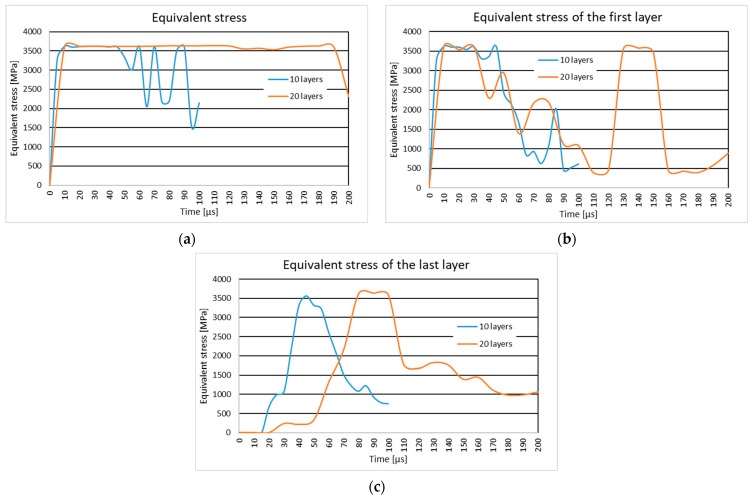
Equivalent stress distribution of the 10-layer fabric panel and the 20-layer fabric panel impacted by the 9 mm Luger FMJ RN 9 mm projectile with an initial impact velocity of 436 m/s. (**a**) Equivalent stress distribution of the panel. (**b**) Equivalent stress distribution of the first layer. (**c**) Equivalent stress distribution of the last layer.

In Figure 11, the equivalent stress distribution is given the during impact of the layered panel impacted by the 9 mm Luger FMJ RN projectile with an initial impact velocity of 436 m/s and the equivalent stress distribution on the first and last layer of the 10-layer panel and the 20-layer fabric panel.

The 20-layer panel is partially penetrated, the projectile is trapped in the fabric, and the 10-layer panel is completely perforated by the projectile. The time moment, $t=1\times 10^{-5}\;s$, records longitudinal stress waves along the entire length of the main yarns.

The time moment, $t=4\times 10^{-5}\;s$, shows that the longitudinal stress waves for the 10-layer panel are distributed over all the yarns up to the area where they are embedded. The 20-layer panel shows stress waves in and around the impact zone, but not up to the ends of the yarns.

### 3.3. The Results of the Influence of Projectile Type

#### 3.3.1. Results of the Simulation of the Ten-Layer Fabric Panel Impacted by a 9 mm Luger FMJ RN Projectile with an Initial Impact Velocity of 398 m/s and a .357 Mag JSP Projectile with an Initial Impact Velocity of 398 m/s

**Figure 12:**
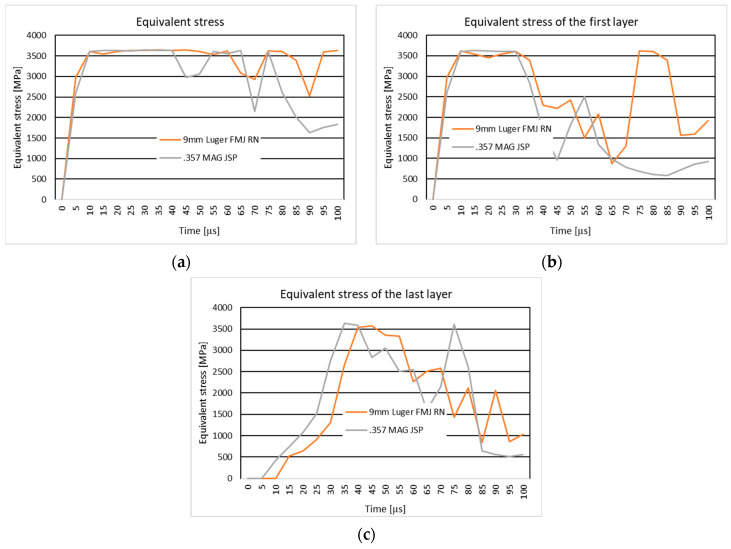
Equivalent stress distribution of the ten-layer fabric panel impacted by the 9 mm Luger FMJ RN projectile with initial impact velocity of 398 m/s and the .357 Mag JSP projectile with initial impact velocity of 398 m/s. (**a**) Equivalent stress distribution of the panel. (**b**) Equivalent stress distribution of the first layer. (**c**) Equivalent stress distribution of the last layer.

In Figure 12, the equivalent stress distribution during the impact of the layered panel impacted by the 9 mm Luger FMJ RN projectile and the .357 Mag JSP projectile with an initial impact velocity of 398 m/s, as well as the equivalent stress distribution on the first and last layer, is given.

The textile panel impacted by a 9 mm Luger FMJ RN projectile with an initial impact velocity of 398 m/s is noted with the letter A and a .357 Mag JSP projectile with initial impact velocity of 398 m/s is noted with letter B.

At the first simulation time, $t=1\times 10^{-6}\;s$, the longitudinal stress waves are distributed on the first layer up to the end of the main yarns for case B. The longitudinal stress waves in case A are distributed in and around the impact zone. Transverse stress waves are visible on the first three layers in case A and on five layers in case B.

In case B, at the time moment, $t=4\times 10^{-5}\;s$, all the layers are broken. Due to the compression of the upper layers and the stretching of the last layers, the break is not central, but is on one side. In case A, the rupture is recorded at the time moment, $t=4.5\times 10^{-5}\;s$, in the middle of the panel. In both cases, the tip and the body of the projectile are not out of the fabric.

In case A, the projectile pushes the broken yarns sideways, while in case B, the broken fragments in the impact zone remain caught on the projectile tip. The projectile changes its trajectory significantly in case B, which also caused the break on one side instead of in the middle, but it is not as deformed as in case A, and it passes through the fabric faster.

#### 3.3.2. Results of the Simulation of the Ten-Layer Fabric Impacted by a 9 mm Luger FMJ RN Projectile with an Initial Impact Velocity of 436 m/s and a .357 Mag JSP with an Initial Impact Velocity of 436 m/s

**Figure 13:**
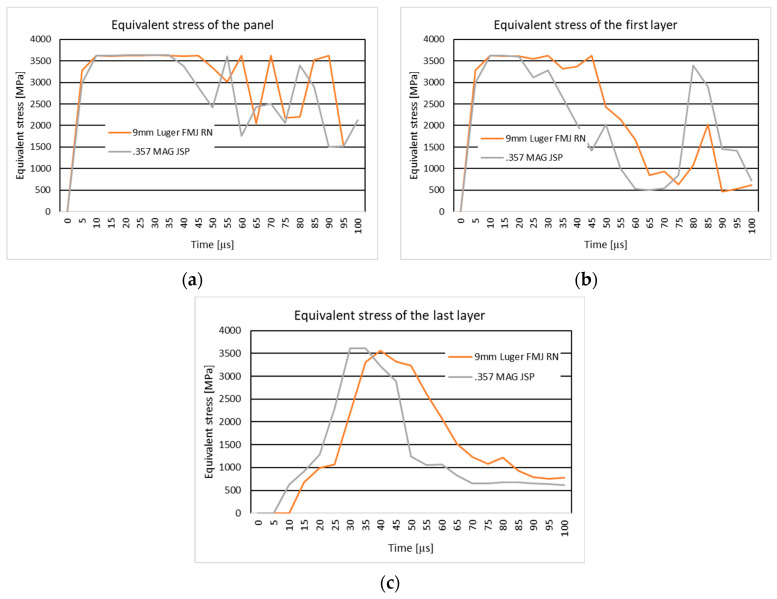
Equivalent stress distribution of the ten-layer fabric panel impacted by 9 mm Luger FMJ RN projectile with initial impact velocity of 436 m/s and the .357 Mag JSP projectile with initial impact velocity of 436 m/s. (**a**) Equivalent stress distribution of the panel. (**b**) Equivalent stress distribution of the first layer. (**c**) Equivalent stress distribution of the last layer.

In Figure 13, the equivalent stress distribution is given during the impact of the layered panel impacted by the 9 mm Luger FMJ RN projectile and 357 Mag JSP projectile with an initial impact velocity of 436 m/s, as well as the equivalent stress distribution on the first and last layer.

The textile panel hit by a 9 mm Luger FMJ RN projectile with an initial impact velocity of 436 m/s is noted with the letter C, and the .357 Mag JSP projectile with an initial impact velocity of 436 m/s is noted with the letter D.

At the second time moment of the simulation, the stress concentrators are also visible outside the impact zone, in case C, on the edge of the two-layer yarns, in the weft direction along their length. The transverse stress waves are visible on several layers. In case D, the stress concentrators are visible in the impact zone on the compressed layers, but also along the length of the main weft-direction yarn on layer 2.

At the time moment, $t=5\times 10^{-5}\;s$, the projectile is highly deformed, showing jacket tearing, which in case D did not happen. The projectile is only deformed. At the end of the simulation, the projectile in case C has its jacket broken.

**Figure 14:**
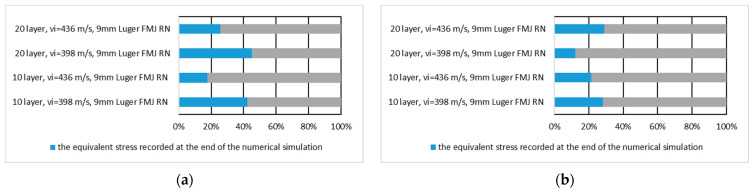
The value of the equivalent stress recorded at the end of the numerical simulation, expressed as a percentage of the maximum stress recorded during the numerical simulation on the whole panel: (**a**) first layer of the panel impacted by the 9 mm Luger FMJ RN projectile; (**b**) last layer of the panel impacted by the 9 mm Luger FMJ RN projectile.

Figure 14 shows the equivalent stress values recorded at the end of the numerical simulation expressed in percent of the maximum stress recorded during the numerical simulation on the whole panel for the first layer of the panel hit by the 9 mm Luger FMJ RN projectile and for the last layer of the panel hit by the 9 mm Luger FMJ RN projectile.

**Figure 15:**
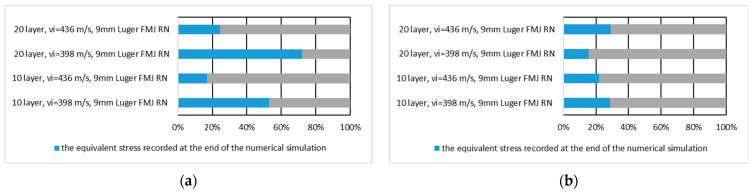
The value of the equivalent stress recorded at the end of the numerical simulation, expressed as a percentage of the maximum stress recorded during the numerical simulation on the layer: (**a**) first layer of the panel impacted by the 9 mm Luger FMJ RN projectile; (**b**) last layer of the panel impacted by the 9 mm Luger FMJ RN projectile.

Figure 15 shows the equivalent stress value recorded at the end of the numerical simulation of the first and the last layer expressed as a percentage of the maximum stress recorded during the numerical simulation on the first layer of the panel hit by the 9 mm Luger FMJ RN projectile and the last layer of the panel hit by the 9 mm Luger FMJ RN projectile.

**Figure 16:**
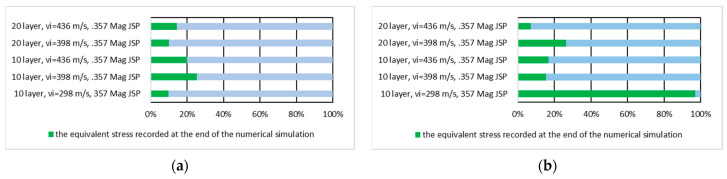
The value of the equivalent stress recorded at the end of the numerical simulation expressed as a percentage of the maximum stress recorded during the numerical simulation on the whole panel: (**a)** first layer of the panel impacted by the .357 Mag JSP projectile; (**b**) last layer of the panel impacted by the .357 Mag JSP projectile.

Figure 16 shows the equivalent stress values recorded at the end of the numerical simulation expressed in percent of the maximum stress recorded during the numerical simulation on the whole panel for the first layer of the panel hit by the .357 Mag JSP projectile and for the last layer of the panel hit by the .357 Mag JSP projectile.

**Figure 17:**
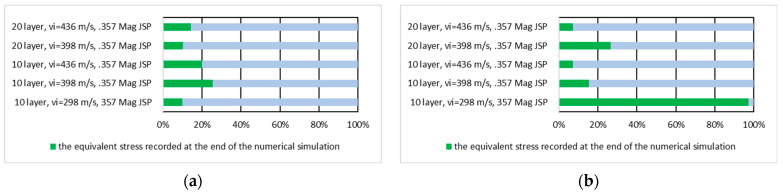
The value of the equivalent stress recorded at the end of the numerical simulation expressed as a percentage of the maximum stress recorded during the numerical simulation on the layer: (**a**) first layer of the panel impacted by the .357 Mag JSP projectile; (**b**) last layer of the panel impacted by the .357 Mag JSP projectile.

Figure 17 shows the equivalent stress value recorded at the end of the numerical simulation of the first and the last layer expressed as a percentage of the maximum stress recorded during the numerical simulation on the first layer of the panel hit by the .357 Mag JSP and the last layer of the panel hit by the .357 Mag JSP.

### 3.4. The Variation of Projectile Velocity During Impact

**Figure 18:**
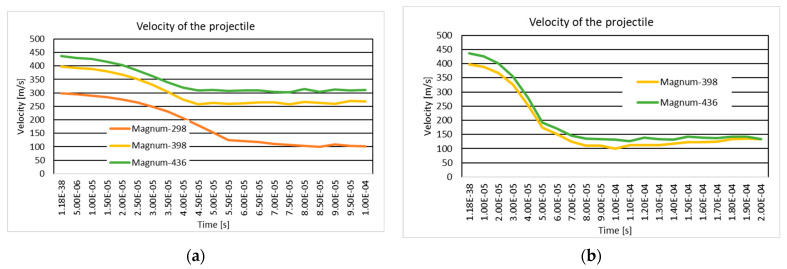
Velocity of the projectile: (**a**) 10-layer panel impacted by .357 Mag JSP; (**b**) 20-layer panel impacted by .357 Mag JSP.

**Figure 19:**
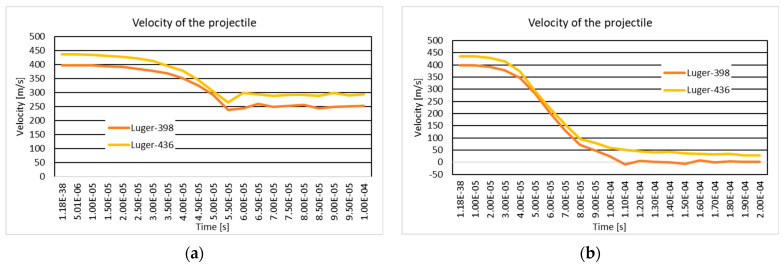
Velocity of the projectile: (**a**) 10-layer panel impacted by 9 mm Luger FMJ RN projectile; (**b**) 20-layer panel impacted by 9 mm Luger FMJ RN projectile.

Figure 18 and Figure 19 show the .357 Mag JSP projectile velocity and 9 mm Luger FMJ RN projectile velocity during the impact on the 10-layer and 20-layer aramid fabric panels. In the case of the ten-layer aramid fabric panel impacted by the .357 Mag JSP projectile, three initial impact velocities (298 m/s, 398 m/s, and 436 m/s) are analyzed. In NIJ Standard-0123.00, if the panel is impacted by the .357 Mag JSP projectile with an initial velocity of 436 m/s and the .357 Mag JSP projectile does not show complete penetration (complete tear of the layer, i.e., the projectile does not exit the fabric), then the panel offers protection for the specific protection level. The velocity of 398 m/s was chosen because, for the 9 mm Luger FMJ RN projectile, the initial impact velocity according to the NIJ Standard-0123.00 [45] for the protection level, NIJ HG1, is 398 m/s. The velocity of 298 m/s was chosen to analyze how the fabric behaves at a lower impact velocity.

## 4. Discussion

### 4.1. Analysis of the Influence of Projectile Velocity

#### 4.1.1. The Ten-Layer Panel Impacted by a 9 mm Luger FMJ RN Projectile with Two Different Impact Velocities: 398 m/s and 436 m/s

The results indicate that a higher velocity leads to a higher stress concentration in the impact zone and rapid propagation of stress waves. Increasing the impact velocity results in accelerated energy absorption and quicker rupture of the fabric. At a velocity of 436 m/s, the propagation of longitudinal stress waves cause the layers to rupture rapidly, indicating that the fabric is unable to redistribute energy efficiently. In contrast, at 398 m/s, the propagation of stress waves is slower and more distributed, allowing for the layers to last longer before failure. The more pronounced projectile deformation at higher velocity may have implications for the design of protective materials, suggesting the need to increase the number of layers. The results are consistent with studies in the literature [24,53,86,87,88,89,90,91], which demonstrate that the impact of velocity also directly affects the mode of breakup and the energy absorption efficiency. The ballistic impact of flexible fabric panels presents a complicated problem.

#### 4.1.2. The Ten-Layer Fabric Panel Impacted by the .357 Mag JSP Projectile with Two Different Impact Velocities: 298 m/s and 436 m/s

The ballistic impact analysis of a ten-layer textile panel demonstrates the effect of projectile velocity on the propagation of stress waves and the mode of layer rupture. Longitudinal and transverse wave propagation are significantly influenced by the impact of velocity, a phenomenon observed in the literature [16]. At higher velocities, transverse waves propagate faster and affect more layers, leading to extensive material destruction.

According to recent studies, thread-level (mesoscopic) modeling and finite element analysis are essential for understanding the mechanisms of stress rupture and propagation in fabrics [62]. This research emphasizes the critical role of projectile velocity in the generation and distribution of stress waves and in the initiation of breakage. These results are confirmed by [16,70,92,93,94].

#### 4.1.3. The 20-Layer Fabric Panel Impacted by a 9 mm Luger FMJ RN Projectile with two Different Impact Velocities: 398 m/s and 436 m/s

In the 398 m/s scenario, the projectile becomes trapped within the fabric layers, demonstrating the panel’s ability to absorb and dissipate energy without complete perforation. Conversely, at 436 m/s, the projectile fully penetrates all layers, with its nose emerging from the fabric, indicating that the fabric’s resistance is insufficient to stop the higher kinetic energy.

The maximum stress values in the initial moments are similar, but the 436 m/s im-pact leads to more significant fabric deformation over time. This is attributed to the greater kinetic energy generating higher stress and inducing severe structural failure earlier. The final recorded stress is notably higher in the 436 m/s case, demonstrating the fabric’s limited capacity to handle elevated energy levels.

The analysis highlights the critical role of impact velocity in determining the extent of fabric deformation and projectile penetration. Lower velocities allow for the fabric to dissipate stress more effectively, preventing perforation, while higher velocities overwhelm the fabric’s structural integrity, leading to complete failure. These findings align with ballistic fabric performance theories, where energy absorption and wave dispersion are key to resisting projectile penetration [16].

#### 4.1.4. The 20-Layer Fabric Panel Impacted by the .357 Mag JSP Projectile with Two Different Impact Velocities: 398 m/s and 436 m/s

Both scenarios exhibit projectile deformation, with the projectile experiencing a significant trajectory change by the end of the simulation. In the 398 m/s case, the projectile rotates nearly 90 degrees, pushing broken yarn fragments along its rotation path. This de-formation behavior mirrors findings in ballistic studies that indicate velocity-driven projectile instability, as highlighted in works like [95], where increased velocity leads to complex interactions between the projectile and the fabric layers, altering the trajectory and increasing structural damage.

### 4.2. Analysis of the Influence of the Number of Layers

#### 4.2.1. The 10-ply Fabric Panel and the 20-ply Fabric Panel Impacted by the .357 Mag JSP Projectile with an Initial Impact Velocity of 436 m/s

In both panels, longitudinal and transverse stress waves play a crucial role in distributing the impact energy throughout the fabric. The 20-layer panel exhibits more wide-spread wave propagation, especially involving both main and secondary yarns, compared to the 10-layer panel, which concentrates stress primarily on the main yarns. This broader distribution in the 20-layer panel helps in dissipating the impact energy more evenly, resulting in reduced localized damage and slower overall failure progression.

This behavior is consistent with [95]’s theory of stress wave dispersion in high-performance fabrics, which suggests that an increased number of layers enhances energy dissipation, leading to a slower peak stress response.

The increased layer count introduces more widespread stress concentrators, not only in the impact zone but also outside it, particularly along main yarns up to the embedded region. This broad distribution of stress suggests that while thicker panels delay failure, they do so by spreading damage over a larger area, which can influence the overall structural stability.

The projectile’s behavior is notably affected by the number of layers. In both cases, the projectile alters its trajectory during penetration, with the angle of deviation being more pronounced in the 20-layer panel. This deviation is a result of the increased interaction between the projectile and the additional fabric layers, which apply greater lateral forces on the projectile, causing rotational instability.

Furthermore, the increased deformation of the projectile in the 20-layer panel under-scores the higher energy absorption capacity of thicker panels. This observation aligns with the findings of [96], who demonstrated that thicker, multi-layered ballistic fabrics induce more projectile deformation due to prolonged contact and friction. These results are also observed in the literature [97,98].

#### 4.2.2. The 10-ply Fabric Panel and the 20-ply Fabric Panel Impacted by the 9 mm Luger FMJ RN Projectile with an Initial Impact Velocity of 436 m/s

The analysis of the 10-layer and 20-layer fabric panels impacted by a .357 Mag JSP projectile at 436 m/s highlights key factors influencing ballistic performance, including stress propagation, energy absorption, structural integrity, and projectile behavior.

In both panels, longitudinal and transverse stress waves play a crucial role in dis-tributing the impact energy throughout the fabric. The 20-layer panel exhibits more wide-spread wave propagation, especially involving both main and secondary yarns, compared to the 10-layer panel, which concentrates stress primarily on the main yarns. This broader distribution in the 20-layer panel helps in dissipating the impact energy more evenly, resulting in reduced localized damage and slower overall failure progression.

The increased resistance and energy absorption observed in the 20-layer panel suggest that it would offer superior protection in real-world applications, particularly against high-velocity threats. However, this enhanced protection comes with trade-offs, such as increased panel weight, material cost, and potential structural compromise due to wide-spread stress dispersal. Balancing the number of layers with the intended level of protection and mobility is essential for optimizing ballistic armor designs.

### 4.3. The Influence of Projectile Type

#### 4.3.1. The Ten-Layer Fabric Panel Impacted by a 9 mm Luger FMJ RN Projectile with an Initial Impact Velocity of 398 m/s and a .357 Mag JSP Projectile with an Initial Impact Velocity of 398 m/s

The study of the impact of two types of projectiles (9 mm Luger FMJ RN and .357 Mag JSP) on a ten-layer fabric panel, both with an impact velocity of 398 m/s, shows that the geometry of the projectile significantly influences the material behavior, even though the impact velocity remains constant.

The 9 mm Luger FMJ RN projectile (with a rounded tip) generates more diffuse stress waves with a more uniform energy distribution in the material, leading to slower and broader penetration. This is consistent with studies [16,98] suggesting that projectiles with rounded tips cause more extensive deformation and slower penetration.

On the other hand, the .357 Mag JSP projectile (with a flat tip) generates more concentrated stress waves, resulting in faster and more direct penetration. These projectiles are more effective at perforating the material and transmit energy more rapidly to the impact zone, leading to faster penetration.

The differences observed in material behavior (deformation, penetration, and fiber rupture) align with previous research, which suggests that flat-tipped projectiles (FMJ) are more efficient at penetrating ballistic materials while round-tipped projectiles (JSP) result in slower but more diffuse penetration.

#### 4.3.2. The Ten-Layer Fabric Impacted by a 9 mm Luger FMJ RN Projectile with an Initial Impact Velocity of 436 m/s and a .357 Mag JSP with an Initial Impact Velocity of 436 m/s

The analysis of the impact of the 9 mm Luger FMJ RN and .357 Mag JSP projectiles on a ten-layer textile panel at an initial impact velocity of 436 m/s reveals significant differences in the behavior of the material, in line with the existing literature [71] in ballistic textile materials and projectile dynamics.

The 9 mm Luger FMJ RN (Full Metal Jacket Round Nose) projectile, with its rounded tip, generates stress waves that are more diffusely distributed across the fabric layers. This is consistent with studies [98,99] suggesting that projectiles with rounded tips tend to disperse energy over a larger area, leading to slower but wider penetration. Such projectiles cause greater deformation across a larger surface area, which aligns with findings from ballistic testing where rounded projectiles result in more diffused damage patterns in materials.

On the other hand, the .357 Mag JSP (Jacketed Soft Point) projectile, with a flatter tip, concentrates the stress at the point of impact, resulting in a more focused and rapid penetration of the fabric. This is in line with the principles of ballistic impact, where flatter-tipped projectiles generate higher localized stress, increasing their penetration potential by transferring energy more efficiently to the material. The results from the .357 Mag JSP projectile indicate that this concentrated stress leads to faster deformation and penetration compared to the 9 mm Luger FMJ RN projectile.

Both projectiles generate longitudinal and transverse stress waves, but the distribution and intensity of these waves differ based on the geometry of the projectile. The 9 mm Luger FMJ RN projectile causes stress waves to propagate through fewer layers, with a more uniform distribution, as observed in Case C. The .357 Mag JSP projectile, on the other hand, creates more concentrated stress in the impact zone and spreads further outside the impact zone, as seen in Case D. This suggests that projectiles with more concentrated energy release, such as the .357 Mag JSP, tend to induce more localized damage, while those with more diffused energy release (like the 9 mm Luger FMJ RN) cause wider but less intense deformation.

Failure mechanisms observed in both cases, such as the rupture of the first layers of fabric and the varying degrees of projectile deformation, are also in line with findings from previous studies on textile armor. Case C, with the 9 mm Luger FMJ RN projectile, shows significant fabric damage, including breaking of the yarns and jacket tearing, which is consistent with the observed slower, more extensive penetration typical of round-nose projectiles. On the other hand, Case D shows a more focused, penetration with less deformation of the projectile, supporting the idea that flat-tip projectiles cause more efficient penetration through materials while maintaining their integrity.

The deformation and failure modes of the fabric panels have high importance in under-standing both the projectile geometry and the material properties in ballistic protection design. Previous work [97,98,100] has demonstrated that while flat-tip projectiles are more effective in piercing through armor, round-tip projectiles often cause more comprehensive damage, spreading the energy over a larger area and resulting in different types of fabric failure.

## 5. Conclusions

The ballistic impact of flexible fabric panels presents a complicated problem which can be analyzed at several scales due to several factors, such as the anisotropic behavior of the materials, the projectile impact velocity, and also the projectile geometry and boundary conditions. The analysis of these factors presents a topic of great interest still today, even though they have been analyzed for decades because the mechanisms occurring during impact are not well understood at smaller scales of analysis.

In this study, 3D finite element (FE) models are analyzed at the yarn-length scale to understand the influence of impact velocity, projectile geometry, and number of layers. The yarn-level analysis shows that the yarn is subjected to stress states (stretching) including transverse compression, axial compression, and transverse shear significant enough to cause breakage during impact. The stress waves lead to kinking in the fiber sufficient to cause compression kinking and, in turn, local fibrillation in the fiber.

The 20-layer panel impacted by the 9 mm Luger FMJ RN FMJ RN 9 mm projectile at 398 m/s demonstrated the ability to completely stop the projectile without perforation, as required by NIJ HG1. In contrast, the ten-layer panel was insufficient, allowing for complete penetration at the same velocity, indicating the need for an increased number of layers to meet the standard.

At impact with the .357 Mag JSP projectile at 436 m/s, both panels failed, indicating that the level of protection provided by these panels is not sufficient to meet the NIJ HG1 requirements under these conditions. This result highlights that projectile velocity and geometry play a critical role in the ballistic performance of the material.

The inclusion of the NIJ HG1 requirements in this study allowed for a clear assessment of the level of protection offered by different textile panel configurations. The results emphasize the importance of designing ballistic equipment in accordance with these standards to ensure effective protection against real threats.

To improve the performance of ballistic panels and meet NIJ HG1 requirements in more demanding scenarios, future research should consider optimizing panel materials and architecture (use of new composite materials or hybrid structures to improve energy dissipation and reduce stress wave propagation), increasing the number of layers (this study demonstrates that more layers provide better protection, but this implies a trade-off between total panel weight and user mobility). Integrating meso-level analysis (advanced fiber-level models could allow for a more detailed understanding of energy absorption mechanisms and the breakage process, providing innovative solutions to increase protection efficiency).

This study underlines the importance of aligning ballistic protection equipment to NIJ HG1 standards and highlights the need for continuous improvement in panel design to cope with increasingly varied projectile types and velocities.

## Figures and Tables

**Table 1:** Protection levels of NIJ Standard-0123.00 [65].

NIJ Ballistic Protection Level	Test Threat	Velocity
NIJ HG1	9 mm Luger FMJ RN	398 m/s
	.357 Mag JSP	436 m/s
NIJ HG2	9 mm Luger FMJ RN	448 m/s
	.44 MAG JHP	436 m/s
NIJ RF1	7.62 × 51 mm M80 Ball NATO FMJ	847 m/s
	7.62 × 39 mm MSC Ball Ammunition	732 m/s
	5.56 mm M193	990 m/s
NIJ RF2	7.62 × 51 mm M80 Ball NATO FMJ	847 m/s
	7.62 × 39 mm MSC Ball Ammunition	732 m/s
	5.56 mm M193	950 m/s
NIJ RF3	30.06 M2 AP	878 m/s

NIJ—National Institute of Justice; HG—handgun threats; RF—representing rifle; FMJ—full metal jacketed; RN—round nose; JSP—jacketed soft point; JHP—jacketed hollow point; MSC—mild steel core; NATO—North Atlantic Treaty Organization.

**Table 2:** The material properties of the yarn.

Property	Value	Unit
Density	1440	Kg/m^3^
Young’s Modulus	95,000	MPa
Poisson’s Ratio	0.35	-
Yield Strength	3600	MPa
Tangent Modulus	1000	MPa
Equivalent Plastic Strain EPS	0.04	-

**Table 3:** The material properties of the projectile core for all case.

Property	Value	Unit
Density	11,340	Kg/m^3^
Young’s Modulus	16,000	MPa
Poisson’s Ratio	0.44	-
Johnson Cook Strength		
Initial Yield Strength	24	MPa
Hardening Constant	300	MPa
Hardening Exponent	1	-
Strain Rate Constant	0.1	-
Thermal Softening Exponent	1	-
Melting Temperature	760	K
Reference Strain Failure	1	(/sec)

**Table 4:** The material properties of the .357 Mag FMJ projectile jacket.

Property	Value	Unit
Density	8670	Kg/m^3^
Young’s Modulus	1.27 × 10^5^	MPa
Poisson’s Ratio	0.32	-
Yield Strength	280	MPa
Tangent Modulus	1150	MPa
Specific Heat	385	J/KgK
Shear Modulus	37,000	MPa
EOS Gruneisen model		
Gruneisen Coeficient	2.04	-
Parameter C1	3726	s m^−1^
Parameter S1	1.434	-
Parameter S2	0	s m^−1^

**Table 5:** The material properties of the 9 mm Luger FMJ RN projectile jacket.

Property	Value	Unit
Density	8300	Kg/m^3^
Young’s Modulus	1.17 × 10^5^	MPa
Poisson’s Ratio	0.34	-
Yield Strength	70	MPa
Tangent Modulus	1150	MPa

**Table 6:** Statistics.

Property	Nodes	Elements
10-layer—.357 Mag JSP	51,465	23,984
10-layer—9 mm Luger FMJ RN	51,948	26,462
20 layer—.357 Mag JSP	101,265	39,984
20 Layer—9 mm Luger FMJ RN	101,748	42,462

## Data Availability

The original contributions presented in this study are included in the article. Further inquiries can be directed to the corresponding author.
